# Ten years of health workforce planning in the Netherlands: a tentative evaluation of GP planning as an example

**DOI:** 10.1186/1478-4491-10-21

**Published:** 2012-08-13

**Authors:** Malou Van Greuningen, Ronald S Batenburg, Lud FJ Van der Velden

**Affiliations:** 1 NIVEL, Netherlands Institute for Health Services Research, PO Box 1568, 3500 BN, Utrecht, The Netherlands

## Abstract

**Introduction:**

In many countries, health-care labour markets are constantly being challenged by an alternation of shortage and oversupply. Avoiding these cyclic variations is a major challenge. In the Netherlands, a workforce planning model has been used in health care for ten years.

**Case description:**

Since 1970, the Dutch government has explored different approaches to determine the inflow in medical schools. In 2000, a simulation model for health workforce planning was developed to estimate the required and available capacity of health professionals in the Netherlands. In this paper, this model is explained, using the Dutch general practitioners as an example. After the different steps in the model are clarified, it is shown how elements can be added to arrive at different versions of the model, or ‘scenarios’. A comparison is made of the results of different scenarios for different years. In addition, the subsequent stakeholder decision-making process is considered.

**Discussion and evaluation:**

Discussion of this paper shows that workforce planning in the Netherlands is a complex modelling task, which is sensitive to different developments influencing the balance between supply and demand. It seems plausible that workforce planning has resulted in a balance between supply and demand of general practitioners. Still, it remains important that the modelling process is accepted by the different stakeholders. Besides calculating the balance between supply and demand, there needs to be an agreement between the stakeholders to implement the advised training inflow.

The Dutch simulation model was evaluated using six criteria to be met by models suitable for policy objectives. This model meets these criteria, as it is a comprehensive and parsimonious model that can include all relevant factors.

**Conclusion:**

Over the last decade, health workforce planning in the Netherlands has become an accepted instrument for calculating the required supply of health professionals on a regular basis. One of the strengths of the Dutch model is that it can be used for different types of medical and allied health professionals. A weakness is that the model is not yet fully capable of including substitutions between different medical professions to plan from a skill-mix perspective. Several improvements remain possible.

## Introduction

Health-care systems are essentially labour-intensive, and so the workforce is an important component for their functioning and performance. Shortages in health-care personnel are a major concern to health policy makers, professional bodies and patient organizations [1-3]. For a long time, the two major challenges in health care worldwide have been to make it less expensive and more capable of meeting the demands of a more accessible, more equitable and more effective health-care delivery system. It is commonly acknowledged that workforce planning is an important instrument for controlling shortage as well as oversupply within the health-care labour market, in particular by determining the inflow in medical training [4-7].

Health-care labour markets in many countries are constantly being confronted with an alternation of shortage and oversupply. This has all the characteristics of what is known in economics as the pork cycle: a cyclical pattern of surplus and shortage as a result of delayed responses to changes in the market. It is a major challenge for policy makers to avoid these cyclic variations between shortage and surplus of health-care personnel. In most countries, such alternations in shortage and oversupply are adjusted by incidental and ad hoc actions that are not able to prevent these variations. Nowadays, these countries are increasingly monitoring the fluctuations with the intention to abolish the pork cycle.

This case study details a simulation model that has been developed since 2000 to support health-care workforce planning in the Netherlands, by calculating the future required inflow in medical specialized training. The goal of this study is to explain the model’s principles, strengths and weaknesses, and to evaluate the extent to which the planning exercise has been accepted by the different stakeholders. Taking the workforce planning of general practitioners (GPs) as an example, the advised and realized inflow over the last 10 years is described, as well as the extent to which the planning process has been successful in reaching a balance between supply and demand.

The case of Dutch GP planning is used to illustrate the working of the model for several reasons. First, GPs play a highly important role within the Dutch health-care system [8], and second, GPs represent one of the largest occupational groups within the physician workforce. GPs administer primary health care 24 hours a day, 7 days a week. In general, patients cannot consult a medical specialist without the mandatory referral by their GP. Most GPs in the Netherlands work in private practices and are self-employed, although a growing number of GPs are being contracted by community health centres. GPs can also be contracted by another (self-employed) GP, or can work as locum GPs. In the Netherlands, there are 60 GPs for every 100 000 inhabitants, which is quite moderate by international comparison [9].

In the Netherlands, GP specialization training lasts three years (full-time) including an internship. The first and third year of training take place at a GP practice, whereas the second year of training consists of six months’ training at a general hospital, three months’ training at a psychiatric hospital and three months’ training at a nursing home. During these three years, GP residents follow one day of training per week at medical school while working in practice the other days [10].

In the next section, the Dutch model for workforce planning will be explained. The Dutch GPs will be used to illustrate the calculations, and the results of the workforce model simulations will be presented for four different years during the last decade. Subsequently, the model will be discussed and evaluated, using six criteria to evaluate simulation models that are developed for policy objectives. At the end of this paper, the conclusions will be presented.

## Case description

### A short history of workforce planning for Dutch health professionals

In the Netherlands, labour market tensions are often on the policy agenda. Shortages in the Dutch health-care workforce become public issues if, for example, people have difficulty finding a GP who registers new patients, or if there are long waiting lists for consulting a medical specialist. Political debates emerge if labour market tensions find expression in a high workload among health professionals, but also in unemployment or underemployment [11-13].

In the Netherlands, workforce planning is considered an important instrument for controlling shortage and oversupply. Before 1972, there was no central planning for health professionals. In those days, this was not seen as a task for the government or the medical professions. Medical schools were autonomous in their decisions on the inflow of students. This situation changed when the *numerus clausus* was introduced in Dutch medical schools in 1972 to limit the oversupply of students and to curb the high costs involved in training physicians. The Ministry of Health, Welfare and Sport advised on this limit and the Ministry of Education, Culture and Science set the limit for the number of students to be enrolled in Dutch medical schools per year. In the 1970s and 1980s, several advisory committees were established to specifically advise the government on the *numerus clausus* threshold for medical schools, mainly to prevent future oversupply of health professionals. In the late 1980s and early 1990s, the government chose to make its own planning models rather than use advisory committees. The planning policy had one aim: maintaining the status quo in the GP workforce and medical specialist workforce density. In this period, cost containment was the main focus of workforce planning. Reaching an adequate supply of physicians to meet the foreseeable demand for care seemed a goal of lesser importance. Such planning was executed at a governmental level until 1992. In this year, the government withdrew from health workforce planning, leaving health workforce planning to the professions. Between 1992 and 1999, professional organizations subsequently conducted their own planning studies. Many professions decided to increase their training capacity, but the government, although still monitoring the health workforce, did not increase the *numerus clausus*.

In the late 1990s, signals of a forthcoming shortage in GPs and medical specialists led to the decision to recentralize the planning of medical specialists. In 1999, three groups of stakeholders (the medical professions, the medical training institutes, and the health insurers) decided to found the Advisory Committee on Medical Manpower Planning (Capaciteitsorgaan). This Advisory Committee is an independent advisory committee which focuses on determining the medical training capacity in the Netherlands required to meet the demand for care. One of its main achievements has been the development of a simulation model. The goal of this model is to measure the current gap between the required and available number of health professionals and the expected balance for the next 10 to 20 years. This should take place through planning the yearly inflow of health professionals in training. Because of the time between the start of initial medical training and entering the labour market for physicians (9 years for GPs, 12 years for medical specialists), the planning model requires projections of developments in the labour market for a period of between 10 and 20 years. The output of the planning model is a calculation of the required yearly inflow in medical training within the next 5–15 years. After these calculations, the results of the model are discussed within specialized platforms of the Advisory Committee on Medical Manpower Planning, which consists of representatives of professionals, health insurers and the medical training institutions [14]. In the following sections, the Dutch simulation model for health professionals will be discussed more extensively [15,16].

### The Dutch simulation model for health professionals

#### *Background*

In the year 2000, the first version of a simulation model was developed to estimate the yearly number of health professionals in training required to meet the future demand for care. This first version of the simulation model was technically developed by the Netherlands institute for health services research (NIVEL: Nederlands instituut voor onderzoek van de gezondheidszorg), which manages this model and executes the calculations. Several sources of information are applied to determine the values for the elements of the model. These sources are based on information about health professionals (e.g. surveys among health professionals, registration databases), about the demand for care (e.g. population projections, expert estimations) and about the training of health professionals (e.g. the number of female students, drop-out rate). A more extensive description of the sources used to estimate each of the elements of the model is shown in Table 1.

**Table 1:** Elements included in the workforce planning model with corresponding data source

**Element**	**Data source**
1 HPs available in baseline year	Registration of HPs
2 Amount of FTE per HP in baseline year	Surveys
3 Available supply (total FTE) in baseline year	Calculation using 1 & 2
4 Unmet demand for care in baseline year	Expert estimations
5 Required supply (total FTE) in baseline year	Calculation using 3 & 4
6 Demographic developments	Population projections and patient registration
7 Required supply (total FTE) in target year	Calculation using 5 & 6 and 19–25 when applicable
8 Outflow	Medical registration, information work status, surveys + unexpected outflow
9 HPs available in target year	Calculation using 1, 8, 10, 11, 12, 13 & 14
10 International migration	Medical registration migration past and expert estimations future migration
11 Labour market return of migration	Information training, medical registration and information work status
12 Number in HP training	Information from HP training
13 Return on training	Information training, medical registration and information work status
14 Labour market return of training	Medical registration and information work status
15 Amount of FTE per HP in target year	Surveys
16 Available supply (total FTE) in target year	Calculation using 9 & 15
17 Difference between available and required supply	Calculation using 7 & 16
18 Required number of HPs in training	Calculation using 17
19 Epidemiological developments	Expert estimations, and empirical data if available
20 Sociocultural developments	Expert estimations, and empirical data if available
21 Change of working hours per FTE	Expert estimations, and empirical data if available
22 Technical developments regarding the profession	Expert estimations, and empirical data if available
23 Developments regarding efficiency	Expert estimations, and empirical data if available
24 Developments regarding horizontal substitution	Expert estimations, and empirical data if available
25 Developments regarding vertical substitution	Expert estimations, and empirical data if available

FTE: full-time equivalent: HP: health professional.

**Figure 1:**
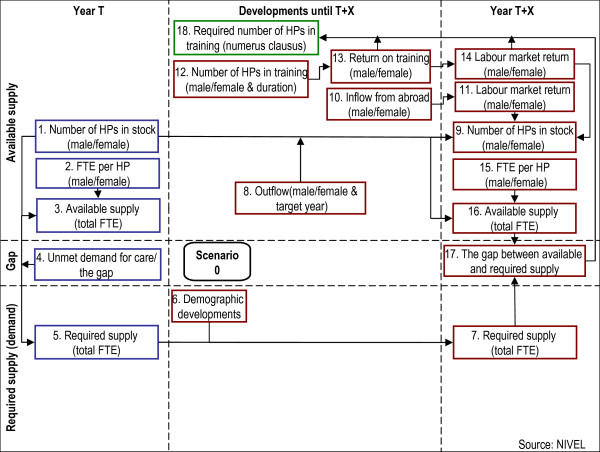
The Dutch simulation model for workforce planning, basic version.

The basic version of the Dutch health workforce planning model is depicted in Figure 1. The 18 elements of this basic version will be explained in the following paragraph. This workforce planning model can be characterized by several classification frameworks of different workforce planning models [17-19]. According to these classification frameworks, the model used in the Netherlands can best be classified as a demand-based model, as the planning of the workforce is not only derived from the inflows and outflows of health professionals, but also by projecting the future demand for a certain occupational group (for example, GPs).

**Figure 2:**
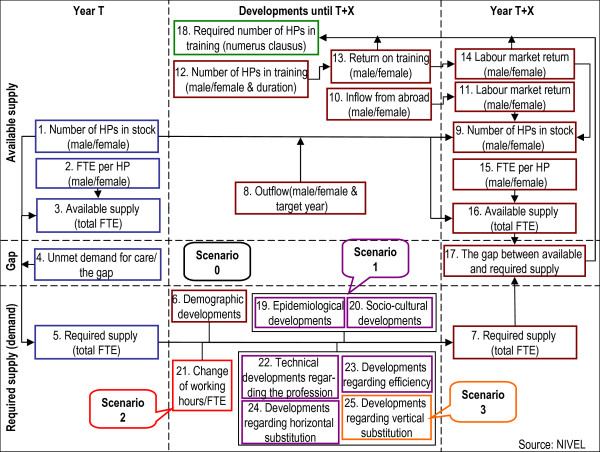
The Dutch simulation model for workforce planning, version including scenarios.

The model and its elements can be divided into three different stages, presented as columns in Figure 1. The elements on the left-hand side of the model refer to the current situation (baseline year, year T). The elements on the right-hand side deal with the target year (year T + X), the projection or target year in the future. The goal of the model is to reach a balance between health-care supply and demand in the target year. Between the baseline year and the target year, in the central part of the figure, the expected developments are represented (between years T and T + X). Calculations of the available and required health care with regard to a specific health profession are compared to estimate the difference between supply and demand in the present and in the future (i.e. the size of a surplus or shortage of health professionals). In Figure 1, the supply side of the model is presented in the upper part of the figure and the demand elements in the lower part. The difference between supply and demand is translated into advice about the required number of health professionals in the first year of training. Below, we present the subsequent steps of the simulation model, referring to the numbers of the elements used in Figures 1 and 2 between brackets.

#### *Description of the baseline model*

##### Step 1: Calculating the current situation (left-hand side of the model)

First, the total available full-time equivalent (FTE) supply (indicated as element 3 in Figure 1) is calculated by computing the product of the total number of health professionals available (element 1) and the amount of FTE (one FTE working on a 100% full-time basis) per health professional (element 2). Both elements are specified by gender (male/female). The gap between supply and demand in the baseline year (‘unmet demand’; element 4) is estimated by experts (see next section) and is used to calculate the required FTE supply of health professionals in the baseline year (element 5). In Table 2, the calculations of this step are illustrated by using Dutch GPs as an example.

**Table 2:** Example of step 1 of the baseline model (scenario 0): General Practitioners in the Netherlands

**Part of model**	**Calculation**
Current available supply	For 2009, the total number of available GPs was 10,215; of these, 6,129 were male and 4,086 were female. On average, male GPs worked 0.822 FTE and female GPs 0.551 FTE. With these numbers, the total available supply in FTE for 2009 can be calculated as 7,290 FTE.
Current required supply	Experts estimated that in 2009 the gap between health-care demand and available supply was 1%. Based on the total available supply in 2009 of 7,292 FTE and the gap of 1%, the required health-care supply can be calculated for 2009; this is estimated at 7,363 FTE.

There are several challenges in finding the information that is needed to calculate the value of each element. One such challenge concerns element 1, which requires information about the number of active health professionals. All medical specialists and GPs who want to practice in the Netherlands are registered by the Medical Registration Committee. However, not all registered specialists are active and this information is sometimes difficult to recover. For Dutch GPs, this information is available as NIVEL has managed its own GP registration since 1975. For other professions, surveys are needed to obtain this information. Another challenge is to obtain information about the average percentage of FTE that health professionals work (element 2). This is mostly obtained from surveys among a representative sample of health professionals. This information is necessary to calculate the value of element 2. The unmet health-care demand in year T (element 4) is estimated by experts, which is a challenge for its high uncertainty ranges. This estimation is partly based on information on waiting lists and job vacancies, but predominantly by the experience of the experts, which makes it vulnerable to subjective interpretation and expectations.

##### Step 2: Developments between baseline year and target year (mid column of the model)

The second step is to estimate the supply and demand for the target year. To estimate the values of the supply and demand for the target year, the values of the elements in the period between the baseline and target year need to be determined. Several elements with regard to changes in supply and demand are considered in this mid column (see Figure 1). See also Table 1 for an overview of the data sources that are used in the Netherlands to determine the values of the elements.

##### *Demographic developments (element 6)*

A key element on the demand side of the model (lower part of Figure 1) is in regards to demographic developments in the period between the baseline year and the target year. These developments represent changes in the age and gender structure of the population. For most health professions, the changes in age structure are the most influential demographic developments in the nearby future on the demand side: the relative size of older groups is increasing while the younger groups are becoming smaller. Older people tend to have a higher demand for care, and therefore this change may lead to an increase in the total demand for health care and the required supply of health professionals. This element is based on population projections from Statistics Netherlands (Centraal Bureau voor de Statistiek) combined with information about the number of contacts with health professionals for different age groups. Based on age and gender, demographic extrapolations have been made using the current health-care consumption per inhabitant and the predicted number of inhabitants for a specific target year. In the extended model, discussed below, a number of other developments are described that also contribute to the total required supply of health professionals.

##### *Outflow (element 8)*

On the supply side of the model (the upper part of Figure 1), the outflow of health professionals in the period between the baseline year and the target year is an important predictor of the future number of health professionals available in year T + X. Reasons for leaving the profession are mostly (early) retirement or choosing another profession. The pattern of retirement is therefore largely determined by the age structure of active health professionals [20]. Furthermore, it is known that most female health professionals tend to leave the profession at an earlier age than males [21].

##### *Health professionals trained abroad (element 10)*

Another contributing factor in the future number of health professionals is the inflow from other countries. However, although the free movement of employees within Europe has been officially regulated since 1985 [22], the inflow from abroad is relatively small for most medical professions. Moreover, some health professionals that have been trained abroad and that come to the Netherlands to work are actually Dutch doctors, who finished medical school in the Netherlands but specialized in another country. The majority of such health professionals followed specialized training in Belgium [23] and return to the Netherlands to occupy a position [8,21,24]. This implies that the inflow of non-Dutch health professionals is low compared with other countries, such as the UK [22].

##### *Training (elements 12 and 13)*

The number of health professionals available in the target year is predicted using different data, including the expected inflow into the specialized training in different years. Attention is paid to the number of women in training in this element, so that feminization of the future workforce can be estimated.

The duration of and return on training are important elements in the model, as they determine the number of graduates and when these graduates enter the labour market [21].

In Table 3, the calculations regarding these developments are illustrated with Dutch GPs*.* From 10 years of experience, the challenges regarding the measurements for Step 2 of the model can be summarized as follows. First, to calculate the percentage of health professionals that will have left practice before the target year, i.e. Element 8 of the model, information is required about the age, career ambition and work status (active or not) of health professionals. Surveys are used to acquire this information, but the retirement expectations of professionals might not always be reliable, in particular if they are at the beginning of their career. For Dutch GPs, the advantage is that this information can be obtained from survey data and the NIVEL GP registration system.

**Table 3:** Example of step 2 of the baseline model (scenario 0): General Practitioners in the Netherlands

**Part of model**	**Calculation**
There are different developments regarding the available supply of GPs in the Netherlands between 2009 and 2019 that will influence the available supply in 2019:	
Outflow	It is estimated that 38.2% (2,341) of male GPs and 19.2% (785) of female GPs working in 2009 will stop work before 2019. This estimation is mostly based on the GPs’ age structure.
Inflow from abroad	It is assumed that between 2009 and 2019, 109 GPs will come from abroad to work in the Netherlands, 46 of whom will be female. It is estimated that 93 of these GPs will still be active in the Netherlands in 2019.
Inflow from training	In the baseline year (2009), there were 1,507 GPs in training, of which 71% were female. The return on training is 98% and therefore 1,477 students from this year will complete their training before 2019. In 2019, 1,320 of them will still be working as GPs. In 2009 and 2010, 1,228 students will start GP training, of which 1,153 will complete their training before 2019. In 2019, 1,054 of these will still be working as GPs. To obtain a complete picture of the size of the inflow from training until 2019, five more years of students, from 2011 to 2016, will have to be taken into account. This means an additional number of 3,070 students will start GP training, of whom 2,883 will graduate before 2019. In 2019, 2,690 will still be working.
Demand developments	It is estimated that the required supply (or health-care demand) will increase by 6.0% as a result of demographic developments in this period.

A second challenge is to achieve a reliable estimation by expert groups on the future number of foreign trained health professionals. Although some information can be used about past international migration, available from the Medical Registration Committee, this still remains a difficult element to estimate as it strongly fluctuates with the labour market conditions in other countries. Finally, collecting reliable information from training institutions on their return on training, i.e. Element 13, can be complicated. This element is calculated as a percentage based on two kinds of information: the number of students starting training and the number of students successfully finishing training. However, training institutions differ in defining these inflow and outflow numbers, due to differences in starting dates and switching behaviour of students within and between training institutions.

##### Step 3: Calculating the future situation (right-hand side of the model)

The right-hand side column in the model (Figure 1) represents the situation in the target year (T + X). First, the expected total available supply of FTE in the future (element 16) is calculated by multiplying the predicted number of health professionals available (element 9) with the predicted percentage of FTE per health professional (element 15). The number of health professionals available is calculated using several data, including the number of health professionals in the baseline year (element 1) and the outflow of health professionals in the intervening years (element 8). In addition, the future number of available health professionals is influenced by the return on training (element 13), the labour market return of the training (element 14), and the inflow from abroad (element 11).

The required supply of health professionals in the target year, measured as the total number of required FTE (element 7), calculated using the number of FTE required in the baseline year (element 5), is influenced by demographic developments (element 6).

##### *Labour market return and future capacity (elements 11, 14 and 15)*

Element 14, in year T + X in the right column of the model, represents the so-called labour market return of health professionals. This element covers the fact that not all health professionals who complete their specialized training start to work in the intended area of specialization. For some professionals this is a career choice, others cannot find the position they want. For example, from 1974 onwards (the start of specialized GP training), 25% of GPs who finished their training did not start to work in their area of specialization. Most of them started working as physicians in other health-care areas. Since the duration of GP training was changed from two to three years in 1987 and the admission procedure has been altered (application instead of admission by lot), the participation rate has risen [14].

The value of element 11 represents the labour market return on migration. This value is a percentage that is based on past international migration and an estimation of the number of health professionals still active after a certain period of time (based on past information). Element 14 represents the labour market return on training. The value of this element is a percentage that is based on the number of students that successfully finish their training and an estimation of the number of health professionals that are still active after a certain period of time (based on past information).

Finally, element 15 represents the future number of health professional FTEs. Information on the average percentage of FTE that health professionals work and on the FTE percentage they wish to work can be obtained from surveys among a representative sample of health professionals.

In Table 4, the calculations of this third step are illustrated using statistics about Dutch GPs. Many of the measurement challenges that have been described for the previous two steps also apply here. Labour market return on training and immigration can be derived from statistics from the Medical Registration Committee. Still, supplementary surveys are needed, in particular to monitor the average FTEs health professional wish to work in the future. These career estimations are queried by questionnaires, and also change within generations, and are therefore associated with certain levels of uncertainty.

**Table 4:** Example of step 3 of the baseline model (scenario 0): General Practitioners in the Netherlands

**Part of model**	**Calculation**
Future available supply	For 2019, it has been predicted that the total number of GPs available will be 12,246; of these, 5,301 will be male and 6,945 will be female. It is estimated that male GPs will work 0.822 FTE and female GPs will work 0.551 FTE on average. Using these numbers, the total available supply in FTE in 2019 can be predicted as 8,187 FTE.
Developments between 2009 and 2019	This number has been derived from the earlier presented estimations: the number of GPs available in 2009 and 2019, the outflow and inflow of GPs between 2009 and 2019, the return on training, labour market return of training, and the inflow from abroad (see Table 3).
Future required supply	For 2019, it has been predicted that the total required supply is 7,807, based on the required supply in 2009 (7,365 FTE, including unmet demand), the estimated gap between supply and demand, and demographic developments until 2019 (which will increase demand by 6.0%).

##### Step 4: Calculating the gap

Step 4 is the final step of the model simulations. The difference between the required supply and the available supply in the target year constitutes the expected gap between supply and demand (element 17). The target of the simulation model is to reach equilibrium by adjusting the future number of health professionals in training (element 18). See Table 5 for the illustration using Dutch GPs.

**Table 5:** Example of step 4 of the baseline model (scenario 0): General Practitioners in the Netherlands

**Part of model**	**Calculation**
Difference between supply and demand	As seen earlier, the total required supply in 2019 is 7,807 FTE, while the total available supply in 2019 is 8,187 FTE. This means that if the baseline model is applied, there will be an oversupply in 2019 of 380 FTE.
Future inflow in training	To correct this oversupply, the model calculates that the future number of GPs in training per year should be decreased from 614 to 476.

#### *Description of the extended model: adding elements to create different scenarios*

The previous four steps showed how the baseline model (scenario 0) for workforce planning is built. In 2001 and 2004, other elements were added to the baseline model to compose several extended versions of the model. The basic model predicts the future gap between supply and demand of health professionals by including only demographic factors to predict the future demand. New scenarios have been developed to extend the modelling of the future demand for health professionals to improve the model’s fit with reality.

Figure 2 depicts what elements have been added to the basic version of the model (scenario 0) on the demand side (lower part), to create the three different scenarios 1, 2 and 3. In practice, these elements are estimations (in terms of a percentage of change between the baseline year and the target year) made by expert groups consisting of representatives of three stakeholder groups: professionals, health insurers and training institutions. The experts base their estimations on information from several sources and on their own experiences. Expert discussions regarding the estimation of the scenario elements are organized in several rounds and on several occasions, not via a predetermined route.

The outputs of the basic and the extended model are calculated in the same way; it is the additional elements that result in the different scenarios explained below.

##### Scenario 1

Scenario 1 adds the influence of epidemiological and sociocultural developments to the model, as well as developments regarding the profession: technical developments, developments in efficiency, and developments regarding horizontal substitution. See Table 6 for the illustration.

**Table 6:** Example of the extended model (scenario 1): General Practitioners in the Netherlands

**Part of model**	**Calculation**
Supply and demand developments	It is assumed that demographic developments will increase the demand for GPs until 2019 by 6.0%, epidemiological developments by 3.0%, sociocultural developments by 5.0%, and technical developments by 1.0%; that developments regarding efficiency will decrease the demand by 2.0%; and that developments regarding horizontal substitution will increase the demand by 5.0%. This leads to the estimation that the required supply in 2019 will be equal to 9,056 FTE.
Difference between supply and demand	As seen earlier, the total available supply in 2019 will be 8,187 FTE; hence, there will be a shortage of 869 FTE GPs if Scenario 1 is applied.
Future inflow in training	To bridge this gap, the future number of GPs in training per year should increase from 614 to 929.

##### *Epidemiological developments (element 19)*

This element represents the changes that take place in the prevalence and incidence of diseases, not related to age and gender, which will continue to increase in the case of some diseases and decrease in others. Lifestyle factors, for instance, influence the incidence of certain diseases. For example, if the percentage of smokers continues to decrease, the number of patients with lung cancer, coronary heart disease, stroke, chronic bronchitis and emphysema is expected to decline. On the other hand, the increasing number of obese people will lead to a rise in the incidence of breast cancer, diabetes mellitus and arthritis [14]. Health statistics published yearly by Statistics Netherlands and the National Institute for Public Health and the Environment are used as a source for this element. The actual value of this element is defined as a yearly change rate in the demand for a health profession, due to these epidemiological developments, between the baseline year and the target year. This percentage is estimated by experts based on the sources mentioned above, including their own expectations.

##### *Sociocultural developments (element 20)*

Element 20 represents sociocultural developments, such as increasing patient empowerment and differences between ethnic groups with respect to their health-care demands. These developments may lead to an increase in the actual demand for health care and, as a result, an expansion of the required supply for health care [14]. The value of this element is defined as the yearly growth rate in demand for health professionals due to these sociocultural developments, which are also determined by expert estimations (Table 1).

##### *Technical developments and developments in efficiency (elements 22 and 23)*

These two model elements represent technical developments regarding the profession and changes in relation to efficiency. Technical developments as a part of the labour process depend strongly on the type of medical speciality and the research and development regarding this medical speciality. The proportional influence of this factor on the future demand for a health profession is estimated by experts, preferable from the specific medical domain or specialty. Medical technical developments can be of great influence on the productivity of health-care services and providers. Many innovations occur in prevention, diagnostics and therapy. Apart from actual developments, there is an increase in the expectations regarding these innovations. As a result of the changing and complicating clinical pictures (multimorbidity) caused by the ageing population, more future technical developments are expected regarding these specific cases. The computerization of the health-care system, which mainly involves supporting processes, is also an area with many innovations.

##### *Developments regarding horizontal substitution (element 24)*

Horizontal substitution refers to the shift of tasks between different professions of the same occupational level, eg shifts between physiotherapists and occupational therapists. Horizontal substitution can occur within larger health-care organizations such as hospitals, but also in primary care.

Professional associations are mostly asked to estimate the value of this element. In addition, information about referrals can be used to determine if activities are (or will be) shifted to other professions and which activities are involved [14,25], causing changes in the future demand for certain health professionals.

During the last 10 years that scenario 1 has been applied in Dutch workforce planning, several challenges have been met. Clearly, elements 19, 20, 22, 23 and 24 are fully dependent on the estimation by expert groups and their ability to forecast and project. To avoid dominance of one interest group, the expert groups consist of representatives of professional associations, training institutions and health insurers. The experts are instructed to base their estimations partly on their own experiences, but also use information from research on specific topics. The decision-making process of these expert groups does not take place via a predetermined route, but has been guided and supported intensively by the Advisory Committee on Medical Manpower Planning during the last years. For the future, a more structured approach to expert consultation will be sought.

##### Scenario 2

After scenario 1 was developed, scenario 2 was introduced in Dutch workforce planning. This scenario adds the change in working hours per FTE (element 21) to the projection of the future demand. This element accounts for the tendency of health professionals to work fewer hours [26]. This scenario element is not based on the labour market behaviour of health professionals but on the system in which they are active. During the last few decades, in many sectors of the Dutch labour market there has been a growing tendency towards reducing the formal working time (working hours-to-FTE ratio), to decrease the strain on the labour market, to increase job quality, and to improve the relationship between work and leisure time. Element 21 is mainly introduced in the simulation model to incorporate this trend and incorporates an estimation of developments that lead to a reduction in working hours, based on the desire of the future generation of health professionals entering the labour market, in particular the female health professionals (Table 7). Expert groups estimate this element as the annual (expected) change in the demand for health professionals, due to a structural reduction in working hours. As addressed above, improving the reliability of this prediction by experts is a key challenge.

**Table 7:** Example of the extended model (scenario 2): General Practitioners in the Netherlands

**Part of model**	**Calculation**
Supply and demand developments	In Scenario 2, the developments of Scenario 1 are repeated and in addition experts assume that the developments of working hours per FTE will neither decrease nor increase the demand for GPs for this period. This means that the estimated required supply in 2019 will be equal to 9,056 FTE (same as in Scenario 1). In contrast, as is depicted in Figure 3, for other years (2000, 2003, 2006) a certain amount of change in working hours per FTE was estimated (Scenario 2).
Difference between supply and demand	As we saw earlier, the total available supply in 2019 will be 8,187 FTE, which means that there will be a shortage of 869 FTE GPs if Scenario 2 is applied (same as in Scenario 1).

##### Scenario 3

The third and last scenario 3 is based on developments regarding vertical substitution (element 25). This element was added to the simulation model in 2006 [27]. Vertical substitution is the shift of activities between health professionals of different professional/educational levels, e.g. shifts between GPs and nurse practitioners. Similar to horizontal substitution, information about referrals and task delegation are used to measure vertical substitution. Experts are informed by this information to estimate the degree to which activities are shifted from one profession to another in different domains of health care (Box 6) [25]. Expert groups estimate this element in terms of the percentage of (expected) annual change in the demand for care for health professions, due to vertical substitution.

The calculations regarding this scenario are illustrated in Table 8 using Dutch GPs.

**Table 8:** Example of the extended model (scenario 3): General Practitioners in the Netherlands

**Part of model**	**Calculation**
Supply and demand developments	In Scenario 3, the developments of Scenario 1 and 2 are repeated and in addition experts assume that the developments regarding vertical substitution will decrease the demand for GPs by 6.0%. It is estimated that the required supply in 2019 will be 8,512 FTE.
Difference between supply and demand	As we saw earlier, the total available supply in 2019 will be 8,187 FTE, which means that there will be an undersupply of 325 FTE if Scenarios 3 is applied for this period.
Future inflow in training	To bridge this gap, the future number of GPs in training per year should increase from 614 to 732.

#### *Advice based on the simulation model for inflow in GP training, 2000–2009*

**Figure 3:**
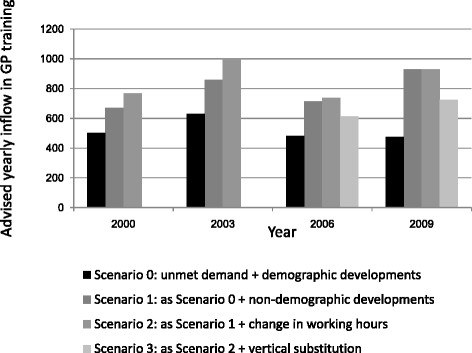
The advised yearly inflow of GPs in training according to different scenarios.

The inflow in the first year of specialized training (the *numerus clausus)* has been adjusted to respond to the developments in the labour market for GPs. Taking the Dutch GP workforce as an example, during the last decade the required inflow in specialized training strongly varied by the different scenarios that were applied. Figure 3 shows the results of the Dutch simulation model as applied at four years during the period 2000–2009. Scenario 0 generates the highest inflow advice in the year 2003, but results in lower inflow numbers advised in the years following. The other scenarios 1, 2 and 3, including expert estimations regarding additional demand developments, have a strong effect on the training inflow advice. For example, between 2000 and 2009, the inflow numbers advised according to scenario 1 were the lowest for 2000 and 2006, but the highest for 2009. In the same period, the inflow advised as generated by scenario 2 were lowest for 2000 and 2006 but the highest for 2003. This is due to the fact that in 2006 and 2009 little or no effect of the change in working hours was expected. In 2006 and 2009, the inflow advice generated by scenario 3 was the lowest, due to inclusion of the (then) expected substitution effect on the demand for GPs [27].

In brief, the figure shows that the results of the scenarios (the advised number of GPs to be trained) mostly increased when adding more developments to the scenarios. An exception is the addition of vertical substitution to scenario 3, which leads to a decrease in the advised number of GPs to be trained.

#### *The policy decision-making process versus the advice*

Since the foundation of the Advisory Committee on Medical Manpower Planning in 2000, the outcomes of the simulation model have become the start of a complex process of decision-making. Within the Dutch workforce planning system for health professionals, the political process of decision-making is an important part [28]. The inflow numbers advised as a result of the scenarios of the simulation model indicate a direction in policy rather than determining the exact number of GPs to be trained, because the different outcomes of the scenarios result in differences in the inflow advised.

When the model simulations, including the different scenarios, have been carried out, the draft inflow recommendations are discussed by the plenary platform of the Advisory Committee. This platform determines the advice to give to the Ministry regarding the yearly inflow in training for health professionals. This advice is subsequently discussed with the Ministry of Health, Welfare and Sport. After the Ministry and national government have decided on the total budget for the training of all (academic) health professionals, this budget steers the numbers that are used to advice medical faculties, schools and universities on their annual student enrolment number.

The decision-making process is complex because different stakeholders with different interests are involved. For example, health insurers are interested in high numbers of medical specialists, as a certain level of oversupply can result in competition and thus may decrease prices. Educators strive to have stable student numbers because teaching capacity is difficult to adjust. Professional organizations often prefer a lower number of medical specialists than insurers because they are averse to too much competition; however, they do want enough young GPs and specialists to take over practices [29].

**Figure 4:**
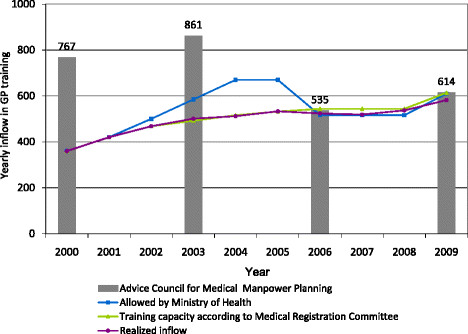
Preferred yearly inflow numbers of different stakeholders into GP training and the realized yearly inflow.

The policy positions about entry numbers in GP training remain a potential point of discussion between stakeholders. Figure 4 depicts what the actual inflow in GP training was in 2000, 2004, 2006 and 2009, compared with the advice based on the model scenarios. The grey bars represent the advice of the simulation model (taken over by the Advisory Committee on Medical Manpower Planning); the different coloured lines represent the inflow allowed by the Ministry, the potential inflow according to medical schools and the Medical Registration Committee, and the realized inflow. As is depicted in the figure, the yearly inflow numbers in training preferred by the various stakeholders are different from the advice generated by the simulation model and theAdvisory Committee. This is particularly the case between 2000 and 2006. Since 2006, when scenario 3 was implemented in the simulation model, the differences in the preferred level of GP training inflow between stakeholders have become smaller. In 2006 and 2009, there seems to be a greater tendency for stakeholders (Ministry, Registration Committee, training institutions) to agree with the advice generated by the simulation model scenarios and the advice of the Advisory Committee.

## Discussion and evaluation

This paper has presented the evolution and structure of the simulation model for health workforce planning in the Netherlands. Data and information about the Dutch GP occupation has been used as an example, to describe the different steps of the simulation model, and to explain the policy process with regard to the preferred yearly inflow in GP training. It has been shown that the Dutch model for health manpower planning is directed at reaching an equilibrium between the supply of and demand for different health professionals. Different scenarios have been developed to improve the fit of the simulation model with reality, and to create a ‘decision space’ for policy makers and stakeholders.

The basic proposition is that health workforce planning controls unintended cyclic labour market fluctuations, and helps to avoid societal waste and loss of quality, both in the consumption and production of health care. Can we conclude from the Dutch experience that this is the case? In the previous sections, we have described that the Dutch model for workforce planning for physicians is accepted by stakeholders and has supported the *numerus clausus* and specialization inflow policy of the Ministry for 10 years. In this respect, the Dutch model is a success. The model is not without complications and challenges, however. Most of these challenges have been described, like the reliability of surveys as data sources, and the need to use mix methods to validate experts’ estimations. Secondly, the relevant question is whether the system of health workforce planning has been successful in ensuring a balance between health supply and demand in the Dutch labour market in the last 10 years. To answer this question for the example of Dutch GPs, we can investigate four indicators to measure labour market balance.

1. The first indicator is that in 2000, the Advisory Committee on Medical Manpower Planning estimated that the unmet demand for GP care was 5%. In 2010, the Advisory Committee states in its report that this unmet demand is close to 0%. Within 10 years, the labour market for GPs has become stable, as there is no national shortage or oversupply of GPs [30].

2. A second indicator is that the number of vacancies is low. According to the weekly magazine of The Royal Dutch Medical Association (Medisch Contact), the number of vacancies over the last years has been stable and relatively low. In 2010 there were 1.7 vacancies per 100 GPs on average [31].

3. The third indicator is that most GPs who complete the specialized GP training find an appropriate place to work. In 2000, 7.2% of active GPs were searching for their desired type of practice. In 2010, this proportion had fallen to 6.5%. According to a survey among practice seekers and locum GPs, these GPs generally found their preferred kind of practice quite quickly in both 2000 and 2010 [32].

4. The fourth and final indicator is the stability of GP density. In 2000, there were 2483 Dutch inhabitants per 1 FTE GP. In 2009, this figure was 2350 inhabitants per FTE [30,33]. Hence, the inhabitant-to-GP ratio is stable and actually increased slightly, which may also reflect that the unmet demand for care has been compensated in the last decade.

Based on these four indicators, it appears that workforce planning of GPs has been successful in sustaining a balance between supply and demand.

Our analysis of the Dutch system also showed that the advice by the Advisory Committee, based on the simulation model, has not always been implemented on a one-on-one basis. Figure 4 shows that, in 2000, the advised inflow in GP training was higher than the inflow that has been realized in that year. Figure 4 also shows, however, that in the following years, the inflow in specialized training for GPs was increasing, and that the gap between the advised and realized number was decreasing. This demonstrates that it takes time for the workforce planning system to adapt to practice, and vice versa. A specific condition in 2000 was that the high advised inflow in GP training in 2000 was used to ‘signal’ to the field an potential upcoming shortage of physicians. To take unexpected developments into account, the workforce planning exercise is carried out approximately every three years for all medical professions. By repeating and adjusting the simulation model and its element values regularly, the planning model can be increasingly in line with actual developments.

In this section of evaluation and conclusion, we finally reflect on the ‘policy value’ of the model. To do so, we apply six criteria that Don and Verbruggen [34] formulated to evaluate models that are designed for policy objectives.

The first criterion they formulated is “qualitative plausibility”. All model elements have to be comprehensible and interpretable in qualitative terms, with the relevant economic theory as the guiding principle. The economic theory applied in the Dutch model of workforce planning is the principle of finding equilibrium between supply and demand in the labour market in the current time and in the future. This assumption is supported by the theories of Abbot [35] and Thurow [36]. Also, the principle of markets being controllable by policy actions is used in this model.

The next three conditions are all supported by the fact that repeated measures registration data provides information regarding several elements in the simulation model. The second criterion (the first of these three conditions) is “quantitative plausibility”. According to this criterion, the elements of the model should have realistic numerical values in the light of stylized facts, input–output ratios and institutional knowledge. Most elements of which the Dutch model is composed are based on information from these types of repeated measures registration data, which are updated regularly. Some elements are an exception to this: these scenario elements are determined by expert estimations.

The third criterion reflects the required “broad correspondence of the model with results of empirical studies”. Estimation results can only be interpreted according to the hypothesis that we know the real model. To acquire information for the elements of the Dutch model, results of repeated and validated studies and surveys are used as illustrated earlier in this paper. The use of this kind of data implies that the results of the model simulations have a close connection with reality.

The fourth criterion represents the need for a “good match with recent data”. The main applications of a model tend to relate to the future, and therefore it is important to use up-to-date information and that the model can describe the recent economic situation, i.e. the starting point of the analysis. For this reason, to keep the data for the Dutch model up to date, results of repeated studies and annual registration data are used, for example from the Medical Registration Committee and the NIVEL GP registration.

The fifth criterion demands “good simulation characteristics” of the model as a whole: plausibility of the equations and their interrelationships, including the analyses of policy scenarios and uncertainty ranges. The equations used in the Dutch simulation model are simple, and most relationships are straightforward and designed close to reality, which is also concluded by Smits et al. [29]. They are, to a large extent, based on demographic information. The extension with scenario elements is numerically simple, because a percentage of change is used to calculate the scenario results. The scenarios are included to show uncertainty. The equations and scenarios have been explained more extensively earlier in this paper.

The sixth condition is the “suitability of the model for the analysis in question”. All the relevant relationships which play a role from the theoretical economic perspective should be incorporated into the model and correctly quantified.

From the six evaluation points, it can be additionally concluded that the Dutch model is a comprehensive model that probably include all relevant factors, while it is also a parsimonious model. It is considered important to find a trade-off between a small and simple model and a large and complex model. A large model would reflect reality in the most complete way, but it may lead to a very complex, and as a result, unstable model. For this reason, the Dutch simulation model actually has to be parsimonious to remain stable. As concluded earlier, the model has been accepted by the different groups of stakeholders, who inspect the model and interpret its outcomes every time new calculations are executed, to advise the Ministry.

## Conclusion

We can conclude that the health workforce planning model that has been in use in the Netherlands for the last ten years, has significant policy value and has been successful in stabilizing the labour market for physicians. In the previous sections, the workforce planning system and the simulation model is broadly described and evaluated. Obviously, there are also other performance measurements for workforce planning and labour market stability. For example, one can opt to do workforce planning based on a normative framework on the skills or competencies that are demanded in health-care services, projecting the ideal skill-mix of health professionals at the organizational level [37,38]. Or one could depart from the notion that misfits as underemployment [11-13] or overeducation [39-41] are to be minimized at the health-labour market, and base workforce planning on this type of goals.

It should be recognized that the current health workforce planning system in the Netherlands aims to achieve a numerical equilibrium in the labour market, taking into account the qualitative fit between supply and demand. In this respect, the planning system and the model are accepted by policymakers and stakeholders. In principle, the model can be used for all types of medical and allied health professionals, as the model is designed as “one size fits all”. Another strength of the model is its flexibility. The elements for non-demographic changes can encompass several types of developments that are modelled as expected percentages of yearly change (delta). This makes it numerically easy for experts to estimate trends in terms of relative changes, not absolute numbers. Thus it is easily expandable with different scenarios by using the different elements that represent demographic and non-demographic developments. Still, several weaknesses of the model have also become clear. The simulation model as such is complex, because it contains many elements, heuristics, submodels and data sources. In addition, the model is not capable of fully simulating the demand and supply of different medical professions in conjunction, i.e. to model systems of profession and training. This is an important weakness of the model, as interactions between health-care professionals are becoming more and more important in view of horizontal and vertical substitution, skill-mix perspectives and the mutual interaction between professional and educational systems in health-care [42]. The fact that workforce planning in the Netherlands occurs at a national level can also be considered a limitation as well. It might become problematic to control regional labour market tensions, such as GPs having difficulty finding successors for their practices.

Other countries that are starting or re-evaluating workforce planning in health care can learn from the strengths and weaknesses of the Dutch model and the experiences as presented in this paper. However, health-care systems and health-labour markets in other countries will certainly deviate from the Netherlands at some or many points. Future international comparative research needs to be conducted into the possibility to adapt the simulation model to these aspects [43].

## Competing interests

The authors declare that they have no competing interests.

## Authors’ contributions

MVG collected information, and drafted and revised the manuscript. LVDV is one of the designers of the original model. RB and LVDV helped draft and revise the manuscript. All authors read and approved the final manuscript.
